# Comparative studies of glycosylphosphatidylinositol-anchored high-density lipoprotein-binding protein 1: evidence for a eutherian mammalian origin for the *GPIHBP1* gene from an *LY6*-like gene

**DOI:** 10.1007/s13205-011-0026-4

**Published:** 2011-10-18

**Authors:** Roger S. Holmes, Laura A. Cox

**Affiliations:** 1 Department of Genetics, Texas Biomedical Research Institute, San Antonio, TX USA; 2 Southwest National Primate Research Center, Texas Biomedical Research Institute, San Antonio, TX USA; 3 School of Biomolecular and Physical Sciences, Griffith University, Nathan, Brisbane, QLD Australia; 4 Department of Genetics, Southwest National Primate Research Center, Texas Biomedical Research Institute, San Antonio, TX 78227 USA

**Keywords:** Mammals, Amino acid sequence, *GPIHBP1*, *LY6*-like, *BCL11A*, Evolution, Chylomicronemia

## Abstract

Glycosylphosphatidylinositol-anchored high-density lipoprotein-binding protein 1 (GPIHBP1) functions as a platform and transport agent for lipoprotein lipase (LPL) which functions in the hydrolysis of chylomicrons, principally in heart, skeletal muscle and adipose tissue capillary endothelial cells. Previous reports of genetic deficiency for this protein have described severe chylomicronemia. Comparative GPIHBP1 amino acid sequences and structures and *GPIHBP1* gene locations were examined using data from several mammalian genome projects. Mammalian *GPIHBP1* genes usually contain four coding exons on the positive strand. Mammalian GPIHBP1 sequences shared 41–96% identities as compared with 9–32% sequence identities with other LY6-domain-containing human proteins (LY6-like). The human N-glycosylation site was predominantly conserved among other mammalian GPIHBP1 proteins except cow, dog and pig. Sequence alignments, key amino acid residues and conserved predicted secondary structures were also examined, including the N-terminal signal peptide, the acidic amino acid sequence region which binds LPL, the glycosylphosphatidylinositol linkage group, the Ly6 domain and the C-terminal α-helix. Comparative and phylogenetic studies of mammalian *GPIHBP1* suggested that it originated in eutherian mammals from a gene duplication event of an ancestral *LY6*-like gene and subsequent integration of exon 2, which may have been derived from *BCL11A* (B-cell CLL/lymphoma 11A gene) encoding an extended acidic amino acid sequence.

**Electronic supplementary material:**

The online version of this article (doi:10.1007/s13205-011-0026-4) contains supplementary material, which is available to authorized users.

## Introduction

Recent studies (Ioka et al. 2003; Beigneux et al. 2007) have shown that a glycosylphosphatidylinositol-anchored high-density lipoprotein-binding protein 1 (GPIHBP1) of capillary endothelial cells is required for the metabolism of triglyceride-rich lipoproteins in mammalian plasma. This glycoprotein binds lipoprotein lipase (LPL) and apolipoproteins (apoA-V) strongly (Gin et al. 2007, 2011) and may serve as a platform for lipolysis within capillaries, particularly in tissues which show high expression levels for both *GPIHBP1* and *LPL* genes, such as heart, skeletal muscle and adipose tissue (Beigneux et al. 2007; Wion et al. 1987; Havel and Kane 2001; Young et al. 2007). Studies of *Gpihbp1*^*−*^*/Gpihbp1*^*−*^ knock out mice have shown that *GPIHBP1*-deficiency causes severe hypertriglyceridemia with very high plasma triglyceride levels of 2,000–5,000 mg/dl (Beigneux et al. 2007; Young et al. 2007).

Human clinical studies have also examined loss of function *GPIHBP1* mutations leading to familial chylomicronemia. Wang and Hegele (2007) reported two siblings with severe chylomicronemia of 160 patients examined exhibiting chylomicronemia who were homozygous for a *GPIHBP1* gene missense mutation (G56R). Franssen et al. (2010) and Olivecrona et al. (2010) have recently identified mutations of conserved cysteines (C65S, C65Y and C68G) in the Ly6 domain of GPIHBP1 in familial chylomicronemia, while Beigneux et al. (2009) have reported a mutant GPIHBP1 (Q115P) which lacked the ability to bind LPL and chylomicrons in a patient with chylomicronemia.

Biochemical studies (Beigneux et al. 2007; Gin et al. 2007, 2011) have suggested that GPIHBP1 is localized on the luminal and abluminal capillary endothelial cell surfaces where it is bound by a glycosylphosphatidylinositol anchor and binds strongly to LPL. GPIHBP1 serves as an LPL transporter from the sub-endothelial spaces to the luminal face of capillaries, enabling lipolysis of circulating triglycerides localized within plasma chylomicrons (Davies et al. 2010; Fisher 2010). Molecular modeling of human GPIHBP1 (Beigneux et al. 2007) and biochemical analyses (Gin et al. 2007) have shown that this protein contains at least four major domains with distinct roles: an N-terminal signal peptide which targets the intracellular trafficking of GPIHBP1 to the cell surface via the endoplasmic reticulum; a very acidic amino acid domain within the GPIHBP1 amino-terminal region may play a role in binding to the positively charged residues of the heparin-binding domain for LPL and apolipoproteins; a cysteine-rich LY6 domain also contributes to LPL binding, as shown by site-directed mutagenesis and human clinical mutation studies (Franssen et al. 2010; Olivecrona et al. 2010); and a C-terminal region which contains a hydrophobic domain which is replaced by a glycosylphosphotidylinositol anchor within the endoplasmic reticulum and which binds GPIHBP1 to the endothelial cell surface (Nosjean et al. 1997; Fisher 2010; Ory 2007). Recently, Gin et al. (2011) have reported several important GPIHBP1-binding properties and have shown specific binding for LPL whereas other related neutral lipases, hepatic lipase (HL) and endothelial lipase (EL), do not bind. In addition, GPIHBP1 also binds APO-A5 strongly whereas another lipid transport protein (APO-A1) does not.

Structures of mammalian *GPIHBP1* genes have been reported in association with a number of mammalian genome sequencing projects, including human, mouse and rat (Mammalian Genome Project Team 2004; Rat Genome Sequencing Project Consortium 2004), and some mammalian GPIHBP1 cDNA and protein sequences have been described (Ioka et al. 2003; Beigneux et al. 2007; Beigneux et al. 2009a, b). Human, mouse and rat *GPIHBP1* genes contain four exons of DNA encoding GPIHBP1 sequences (Thierry-Mieg and Thierry-Mieg 2006).

This paper describes predicted gene structures and amino acid sequences for several mammalian *GPIHBP1* genes and proteins, and predicted secondary structures for mammalian GPIHBP1 proteins. In addition, we examine the relatedness for mammalian *GPIHBP1* with other lymphocyte antigen-6 (*Ly6*-like) genes and proteins, and describe an hypothesis for the origin of the *GPIHBP1* gene within eutherian mammals from an ancestral mammalian *LY6*-like gene and subsequent integration of an exon within the mammalian *GPIHBP1* gene encoding the acidic amino acid LPL-binding platform previously described for human and mouse GPIHBP1 (Beigneux et al. 2007; Gin et al. 2007, 2011).

## Methods

### Mammalian *GPIHBP1* gene and protein identification

Basic Local Alignment Search Tool (BLAST) studies were undertaken using web tools from the National Center for Biotechnology Information (NCBI) (http://blast.ncbi.nlm.nih.gov/Blast.cgi) (Altschul et al. 1997). Protein BLAST analyses used mammalian GPIHBP1 amino acid sequences previously described (Table 1). Non-redundant protein sequence databases for several mammalian genomes were examined using the blastp algorithm, including human (*Homo sapiens*) (International Human Genome Consortium 2001); chimpanzee (*Pan troglodytes*) (Chimpanzee Sequencing and Analysis Consortium 2005); orangutan (*Pongo abelii*) (http://genome.wustl.edu); rhesus monkey (*Macaca mulatta*) (Rhesus Macaque Genome Sequencing and Analysis Consortium 2007), cow (*Bos Taurus*) (Bovine Genome Project 2008); horse (*Equus caballus*) (Horse Genome Project 2008); mouse (*Mus musculus*) (Mouse Genome Sequencing Consortium 2002); rat (*Rattus norvegicus*) (Rat Genome Sequencing Project Consortium 2004); opossum (*Monodelphis domestica*) (Mikkelsen et al. 2007); and platypus *(Ornithorhynchus anatinus)* (Warren et al. 2008). This procedure produced multiple BLAST ‘hits’ for each of the protein databases which were individually examined and retained in FASTA format, and a record kept of the sequences for predicted mRNAs and encoded GPIHBP1-like proteins. These records were derived from annotated genomic sequences using the gene prediction method: GNOMON and predicted sequences with high similarity scores for human GPIHBP1. Predicted GPIHBP1-like protein sequences were obtained in each case and subjected to analyses of predicted protein and gene structures.

**Table 1:** Mammalian *GPIHBP1* and human *LY6*-like genes and proteins

*GPIHBP1* gene	Species	RefSeq ID Ensembl^a^	GenBank ID	UNIPROT ID	Amino acids	Chromosome location	Coding exons	Gene size bps	Subunit MW	Signal peptide (cleavage site)	Gene expression level^f^
Human	*Homo sapiens*	NM_178172	BC035810	Q8IV16	184	8:144,295,143-144,297,390	4 (+ve)	3,976	19,806	1-20 [RG-QT]	0.4
Chimpanzee	*Pan troglodytes*	XP_001151889^a^	–^b^	–^b^	166	8:143,181,557-143,183,786	4 (+ve)	2,230^c^	17,540	1-20 [RG-QT]	na
Orangutan	*Pongo abelii*	XP_002819549^a^	–^b^	–^b^	184	8:151,582,751-151,585,275	5 (+ve)	2,525^c^	19,778	1-20 [RG-QT]	na
Rhesus	*Macaca mulatta*	XP_001085384^a^	–^b^	–^b^	184	8:145,833,092-145,835,237	4 (+ve)	2,146^c^	19,768	1-22 [QA-QQ]	na
Marmoset	*Callithrix jacchus*	XP_002759233^a^	–^b^	–^b^	182	16:51,444,208-51,447,357	4 (−ve)	3,150^c^	19,993	1-22 [QA-EP]	na
Mouse	*Mus musculus*	NM_026730	BC061225	Q9D1N2	225	15:75,427,109-75,428,551	4 (+ve)	1,556	24,566	1-22 [WA-QE]	0.7
Rat	*Rattus norvegicus*	NM_001130547	–^b^	–^b^	236	7:113,538,462-113,540,137	4 (+ve)	1,676^c^	25,562	1-22 [WA-QE]	0.1
Guinea pig	*Cavia porcellus*	ENSCPOT2066^d^	–^b^	–^b^	167	sc95:2379881-2381261^e^	4 (+ve)	1,381^c^	18,240	1-22 [QA-QE]	na
Horse	*Equus caballus*	XP_001496557^a^	–^b^	–^b^	176	9:81,888,709-81,890,489	4 (+ve)	1,781^c^	19,003	1-20 [SG-QV]	na
Cow	*Bos taurus*	XP_590408^a^	–^b^	–^b^	171	14:1,462,446-1,464,219	4 (+ve)	1,774^c^	17,990	1-22 [RA-QE]	na
Dog	*Canis familaris*	XP_851590	–^b^	–^b^	180	13:136,185,964-136,187,786	4 (+ve)	1,482^c^	18,383	1-20 [RA-QD]	na
Pig	*Sus scrofa*	–^b^	CF361073^d^	–^b^	180	4:136,185,964-136,187,786	4 (−ve)	1,823^c^	19,274	1-22 [RA-QE]	na
*LY6*-like gene											
*PSCA*	*Homo sapiens*	NM_005672	BC048808	O46653	123	8:143,748,728-143,761,153	3 (+ve)	2,268	12,912	1--20 [TA-LL]	1.2
*LY6K*	*Homo sapiens*	NM_017527	BC117142	Q17RY6	165	8:143,781,946-143,784,786	3 (+ve)	4,054	18,673	1-17 [WT-DA]	0.9
*SLURP1*	*Homo sapiens*	NM_020427	BC105135	P55000	103	8:143,822,564-143,823,803	3 (−ve)	1,467	11,186	1-22 [EA-LK]	0.1
*LYPD2*	*Homo sapiens*	NM_205545	BC119019	Q6UXB3	125	8:143,831,704-143,833,869	3 (−ve)	2,234	13,115	1-22 [PA-LR]	0.1
*LYNX1*	*Homo sapiens*	NM_177476	BC032036	Q9BZG9	116	8:143,856,588-143,857,375	3 (−ve)	5,823	12,641	1-20 [QA-LD]	1.8
*LY6D*	*Homo sapiens*	NM_003695	BC031330	B2R5F1	128	8:143,865,011-143,863,294	3 (−ve)	1,711	13,286	1-20 [LT-LR]	0.6
*GML*	*Homo sapiens*	NM_002066	BC126336	Q99445	158	8:143,916,217-143,928,261	3 (+ve)	6,250	17,730	1-17 [AA-SA]	<0.1
*LY6E*	*Homo sapiens*	NM_001127213	BC119708	Q16553	131	8:144,102,357-144,103,203	3 (+ve)	3,926	13,507	1-20 [SS-LM]	4.3
*LY6H*	*Homo sapiens*	NM_002347	BC030192	B2RAD2	140	8:144,239,670-144,241,065	3 (−ve)	2,126	14,669	1-25 [HG-LW]	0.7
*GPIBP1*	*Homo sapiens*	NM_178172	BC035810	Q8IV16	184	8:144,295,143-144,297,390	4 (+ve)	3,976	19,806	1-20 [RG-QT]	0.4

GenBank IDs are derived from NCBI http://www.ncbi.nlm.nih.gov/genbank/, Ensembl ID was derived from Ensembl genome database http://www.ensembl.org, UNIPROT refers to UniprotKB/Swiss-Prot IDs for individual proteins (see http://kr.expasy.org), bps refers to base pairs of nucleotide sequences; the number of coding exons are listed, the predicted signal *N*-peptide cleavage site is listed

*RefSeq* The reference amino acid sequence

^a,c^Predicted Ensembl amino acid sequence

^b^Not available

^d^Refers to an expressed sequence tag (EST) sequence encoding pig GPIHBP1

^e^Guinea pig scaffold

^f^From AceView http://www.ncbi.nlm.nih.gov/IEB/Research/Acembly/

Blast-Like Alignment Tool (BLAT) analyses were subsequently undertaken for each of the predicted GPIHBP1 amino acid sequences using the University of California Santa Cruz (UCSC) Genome Browser [http://genome.ucsc.edu/cgi-bin/hgBlat] (Kent et al. 2003) with the default settings to obtain the predicted locations for each of the mammalian *GPIHBP1* genes, including predicted exon boundary locations and gene sizes. BLAT analyses were similarly undertaken for other mammalian *LY6*-like and vertebrate *BCL11A*-like (encoding B-cell CLL/lymphoma 11A) genes and proteins using previously reported sequences for LY6D, LY6E, LY6H, LY6K, LY6NX1, PSCA, SLURP1, GML, LY6D2 and BCL11A in each case (Tables 1, 2, 3). Structures for human, mouse and rat GPIHBP1 genes and encoded proteins were obtained using the AceView website Thierry-Mieg and Thierry-Mieg 2006) (http://www.ncbi.nlm.nih.gov/IEB/Research/Acembly/index.html?human).

**Table 2:** Mouse, cow, opossum and zebrafish LY6-like genes and proteins

*LY6*-*like gene*	Species	RefSeq ID Ensembl^a^	GenBank ID	UNIPROT ID	Amino acids	Chromosome location	Coding exons (strand)	Gene size bps
*Psca*	*Mus musculus*	NP_082492^a^	BC110462	Q9D7U0	123	15:74,545,285-74,547,024	3 (+ve)	2,231
*Slurp1*		NM_020519	BC125244	Q9Z0K7	110	15:74,558,464-74,554,039	3 (−ve)	1,383
*Lypd2*		NM_026671	BC132407	Q9DD23	127	15:74,564,759-74,562,671	3 (−ve)	1,951
*Lynx1*		NP_035968^a^	BC037541	Q9WVC2	116	15:74,583,477-74,578,272	3 (−ve)	673
*Ly6d*		NP_034872^a^	BC022806	Q14210	127	15:74,592,789-74,593,990	3 (−ve)	1,202
*Ly6o*		EDL29447^a^	BC055822	na	119	15:74,602,554-74,609,268	3 (−ve)	6,715
*Ly6k*		NM_029627	BC049723	Q9CWP4	154	15:74,630,417-74,627,298	3 (−ve)	2,510
*Ly6p*		NM_025929	BC116397	Q9CQ11	111	15:74,710,281-74,712,095	3 (−ve)	1,815
*Ly6e*		NM_008529	BC002116	Q99JA5	136	15:74,785,480-74,790,336	3 (+ve)	903
*Ly6i*		NM_020498	BC125390	Q9WU67	134	15:74,810,347-74,813,489	3 (−ve)	3,143
*Ly6a*		NM_010738	BC002070	P05533	134	15:74,825,695-74,828,034	3 (−ve)	2,340
*Ly6c1*		NM_010741	BC010760	Q91XG0	131	15:74,875,445-74,879,260	3 (−ve)	3,107
*Ly6c2*		NM_001099217	BC092082	P09568	131	15:74,938,976-74,942,097	3 (−ve)	3,122
*Ly6f*		NM_008530	BC152856	P35460	134	15:75,099,160-75,102,277	3 (+ve)	3,118
*Ly6h*		NM_011837	BC028758	Q8K356	139	15:75,397,918-75,381,698	3 (−ve)	1,090
*Slurp1*	*Bos taurus*	XP_002692640^a^	na	na	126	14:1,122,649-1,123,776	4 (−ve)	1,128
*LYPD2*		XP_001256661^a^	na	na	128	14:1,127,319-1,129,201	3 (−ve)	1,883
*LYNX1*		NP_001039686^a^	na	na	116	14:1,141,889-1,142,634	3 (−ve)	746
*LY6D*		NP_001069985^a^	na	na	116	14:1,155,662-1,156,820	3 (−ve)	1,159
*GML1*		DAA72886^a^	na	na	154	14:1,219,764-1,228,363	4 (−ve)	8,600
*GML2*		NP_001070493^a^	na	na	154	14:1,258,190-1,265,326	3 (−ve)	7,137
*LY6E*		NP_001039535^a^	na	na	130	14:1,387,941-1,388,689	3 (+ve)	749
*LY6H*		NP_001073104^a^	na	na	140	14:1,449,309-1,450,750	3 (−ve)	1,442
*SLURP*	*Monodelphis domestica*	XP_001381780^a^	na	na	132	3:428,776,701-428,782,624	3 (−ve)	5,924
*LYPD2*		XP_001381786^a^	na	na	237	3:428,806,270-428,813,247	4 (−ve)	6,978
*LYNX1*		XP_001381791^a^	na	na	162	3:428,858,073-428,880,937	3 (−ve)	22,865
*LYNX2*		XP_001381798^a^	na	na	120	3:428,958,198-428,965,110	3 (−ve)	6,913
*LY6D*		XP_001381801^a^	na	na	117	3:428,986,162-428,995,118	3 (−ve)	8,957
*LY6H1*		XP_001373482^a^	na	na	126	3:439,197,554-439,200,063	3 (+ve)	2,510
*LY6H2*		XP_001373600^a^	na	na	141	3:439,414,602-439,420,686	3 (+ve)	6,085
*LYPD6*	*Danio rerio*	NM_001004670	BC081426	Q66IA6	174	9:24,104,899-24,151,695	4 (−ve)	46,797

Ensembl ID was derived from Ensembl genome database http://www.ensembl.org, UNIPROT refers to UniprotKB/Swiss-Prot IDs for individual proteins (see http://kr.expasy.org), bps refers to base pairs of nucleotide sequences, the number of coding exons are listed

*RefSeq* The reference amino acid sequence

^a^Predicted Ensembl amino acid sequence

**Table 3:** Vertebrate BCL11A genes and proteins

Mammalian BCL11A Gene	Species	RefSeq ID Ensembl^a^	GenBank ID	UNIPROT ID	Amino acids	Chromosome location	Coding exons (strand)	Gene size bps
Human	*Homo sapiens*	NM_018014	BC021098	Q9H165	773	2:60,678,303-60,780,633	5 (−ve)	102,331
Orangutan	*Pongo abelii*	XP_002812058^a^	na	na	808	2:50,366,387-50,465,154	6 (+ve)	98,768
Marmoset	*Callithrix jacchus*	XP_002757779^a^	na	na	808	14:46,690,157-46,792,384	6 (+ve)	102,228
Mouse	*Mus musculus*	NM_016707	BC010585	Q9QYE3	773	11:23,978,391-24,072,787	5 (+ve)	94,397
Pig	*Sus scrofa*	XP_003125157^a^	AK231444	na	773	3:74,933,998-75,031,771	5 (+ve)	97,774
Rabbit	*Oryctolagus cuniculus*	XP_002709742^a^	na	na	821	2:125,646,621-125,730,521	4 (+ve)	83,901
Dog	*Canis familiaris*	XP_865536^a^	na	na	773	10:63,737,516-63,836,852	5 (−ve)	99,337
Chicken	*Gallus gallus*	NM_001031031	AJ551441	Q5F459	796	3:1,829,458-1,877,784	3 (−ve)	48,237
Lizard	*Anolis carolinensis*	XP_003216184^a^	na	na	796	276:252,030-507,710^b^	3 (+ve)	255,681
Zebrafish	*Danio rerio*	NP_001035481^a^	na	A2BE84	829	13:26,077,202-26,148,770	3 (+ve)	71,569

*BCL11A* refers to the gene encoding vertebrate B-cell CLL/lymphoma 11A sequences

Ensemble ID was derived from Ensembl genome database http://www.ensembl.org; UNIPROT refers to UniprotKB/Swiss-Prot IDs for individual proteins (see http://kr.expasy.org); bps refers to base pairs of nucleotide sequences; the number of coding exons are listed

*RefSeq* The reference amino acid sequence

^a^Predicted Ensembl amino acid sequence

^b^Refers to scaffold ID

### Predicted structures, properties and alignments of mammalian GPIHBP1 and human LY6-like sequences

Predicted secondary structures for human and other mammalian GPIHBP1 proteins were obtained using the PSIPRED v2.5 website tools [http://bioinf.cs.ucl.ac.uk/psipred/psiform.html] (McGuffin et al. 2000). Other web tools were used to predict the presence and locations of the following for each of the mammalian GPIHBP1 sequences: SignalP 3.0 for signal peptide cleavage sites (http://www.cbs.dtu.dk/services/SignalP/) (Emmanuelsson et al. 2007); NetNGlyc 1.0 for potential N-glycosylation sites (http://www.cbs.dtu.dk/services/NetNGlyc/); and big-PI Predictor for the glycosylphosphatidylinositol linkage group-anchored sites (http://mendel.imp.ac.at/sat/gpi/gpi_server.html) (Eisenhaber et al. 1998). The reported tertiary structure for human CD59 (membrane-bound glycoprotein) (Leath et al. 2007) served as the reference for the predicted human, rat, pig and guinea pig GPIHBP1 tertiary structures, with modeling ranges of residues 62–138, 69–146, 65–141 and 61–139, respectively. Alignments of mammalian GPIHBP1 sequences with human LY6D, LY6E, LY6H, LY6K, LYNX1 and LYPD2 lymphocyte antigen-6-related proteins or with vertebrate B-cell CLL/lymphoma 11A (BCL11A) sequences were assembled using the ClustalW2 multiple sequence alignment program (Larkin et al. 2007) (http://www.ebi.ac.uk/Tools/clustalw2/index.html).

### Comparative bioinformatics of mammalian *GPIHBP1*, vertebrate *LY6*-like and vertebrate *BCL11A* genes and proteins

The UCSC Genome Browser (http://genome.ucsc.edu) (Kent et al. 2003) was used to examine comparative structures for mammalian *GPIHBP1* (Table 1), vertebrate *LY6*-like (lymphocyte antigen-6 complex; Tables 1, 2) and vertebrate *BCL11A* (B-cell CLL/lymphoma 11A) (Table 3) genes and proteins. We also used the UCSC Genome Browser Comparative Genomics track that shows alignments of up to 28 vertebrate species and evolutionary conservation of *GPIHBP1* gene sequences. Species aligned for this study included 4 primates, 6 non-primate eutherian mammals (e.g., mouse, rat), a marsupial (opossum), a monotreme (platypus) and bird species (chicken). Conservation measures were based on conserved sequences across all of these species in the alignments which included the 5′-flanking, 5′-untranslated and coding regions of the *GPIHBP1* gene.

BLAT analyses were subsequently undertaken using the nucleotide sequence for exon 2 of human *GPIHBP1* using the UCSC Genome Browser [http://genome.ucsc.edu/cgi-bin/hgBlat] (Kent et al. 2003) to identify homologs for this exon in the human genome.

### Phylogenetic studies and sequence divergence

Alignments of mammalian GPIHBP1 and vertebrate LY6-like protein sequences were assembled using BioEdit v.5.0.1 and the default settings (Hall 1999). Alignment ambiguous regions, including the acidic amino acid region of GPIHBP1, were excluded prior to phylogenetic analysis yielding alignments of 60 residues for comparisons of sequences with the zebrafish (*Danio rerio*) LY6-like (LYPD6) sequence (Tables 1, 2). Evolutionary distances were calculated using the Kimura option (Kimura 1983) in TREECON (Van De Peer and de Wachter 1994). Phylogenetic trees were constructed from evolutionary distances using the neighbor-joining method (Saitou and Nei 1987) and rooted with the zebrafish LYPD6 sequence. Tree topology was reexamined by the bootstrap method (100 bootstraps were applied) of resampling and only values that were highly significant (≥90) are shown (Felsenstein 1985).

## Results and discussion

### Alignments of mammalian GPIHBP1 amino acid sequences with human LY6-related antigen sequences

**Fig. 1:**
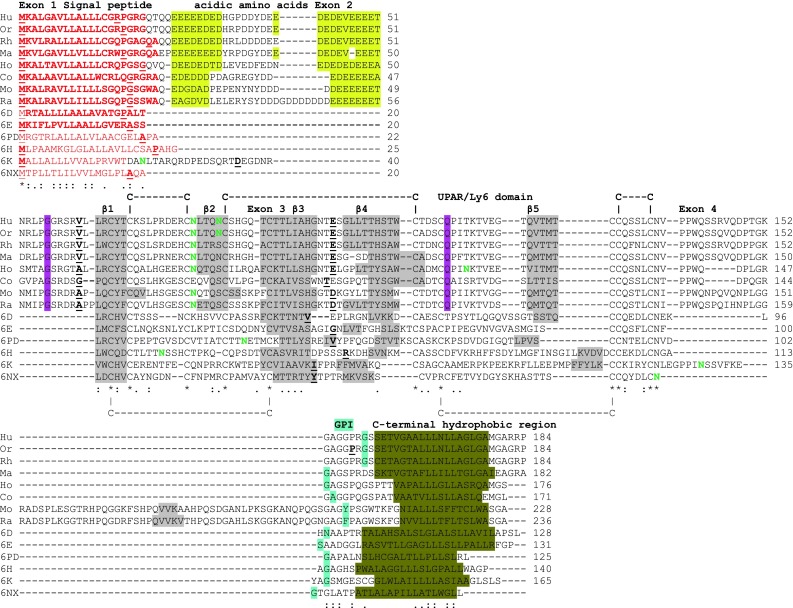
Amino acid sequence alignments for mammalian GPIHBP1 and human LY6-like sequences. See Table 1 for sources of glycosylphosphatidylinositol-anchored high-density lipoprotein-binding protein 1 (GPIHBP1) and human LY6-like sequences: GPIHBP1—*Hu* human, *Or* orangutan, *Rh* rhesus, *Ma* marmoset, *Ho* horse, *Co* cow, *Mo* mouse, *Ra* rat; Human LY6-like: 6D-LY6D; 6E-LY6E; 6D2-LY6D2; 6H-LY6H; 6K-LY6K; 6NX-LY6NX. *Asterisks* show identical residues for proteins, *colon* similar alternate residues, *dot* dissimilar alternate residues. Residues predicted for involvement in N-signal peptide formation are shown in *red*, N-glycosylated and potential N-glycosylated Asn sites are in *green bold*, key GPIHBP1 functional residues 56Gly and 114Gln are in *shaded pink*, predicted disulfide bond Cys residues are shown; α**-**helices predicted for GPIHBP1 are in *shaded yellow*, β-sheets (β1–β5) predicted for mammalian GPIHBP1 or for human LY6-like sequences are in *shadedgrey*, *bold underlined* font shows residues corresponding to known or predicted exon start sites. Exon numbers refer to *GPIHBP1* human gene exons, the sequences for the UPAR/Ly6 domain are shown, C-terminal hydrophobic amino acid segment is shown as *shadedgreen*, known (human and mouse) or predicted mammalian GPIHBP1 and human LY6-like GPI-binding sites are shown in *shadedblue*

The deduced amino acid sequences for orangutan (*Pongo abelii*), rhesus monkey (*Macaca mulatta*), marmoset (*Callithrix jacchus*), horse (*Equus caballus*), cow (*Bos taurus*) and rat (*Rattus norvegicus*) GPIHBP1 are shown in Fig. 1 together with previously reported sequences for human and mouse GPIHBP1 (Beigneux et al. 2007; Gin et al. 2007). In addition, amino acid sequences for several LY6-related lymphocyte antigen sequences are also aligned with the mammalian GPIHBP1 sequences, including human LY6D (Brakenoff et al. 1995), LY6E (Capone et al. 1996), LYPD2 (Clark et al. 2003), LY6H (Horie et al. 1998), LY6K (Ishikawa et al. 2007) and LYNX1 (Mammalian Genome Project Team 2004) (Table 1). Alignments of human and other mammalian GPIHBP1 sequences examined showed identities between 46 and 96%, suggesting that these are the products of the same gene family, whereas comparisons of sequence identities of mammalian GPIHBP1 proteins with human LY6-like lymphocyte antigen sequences exhibited low levels of sequence identities (9–32%), indicating that these are the members of distinct protein families (Table 4).

**Table 4:** Percentage identities for mammalian GPIHBP1 amino acid sequences and the human LY6-like amino acid sequences

GPIHBP1	Human	Orangutan	Rhesus	Marmoset	Mouse	Rat	Guinea pig	Dog	Pig	Cow	Horse	Human	Human	Human	Human	Human	Human
												LY6D	LY6E	LY6H	LY6K	LYPD2	LYNX1
Human	100	96	84	80	54	52	53	60	53	54	59	14	27	26	10	24	13
Orangutan	96	100	85	84	54	53	53	61	54	55	60	17	24	23	10	24	13
Rhesus	84	85	100	73	53	53	51	57	50	49	59	10	30	25	13	24	12
Marmoset	80	84	73	100	46	50	51	57	51	46	57	18	27	22	9	23	12
Mouse	54	54	53	46	100	82	63	53	51	50	55	14	20	22	9	25	14
Rat	52	53	53	50	82	100	61	51	49	52	51	15	20	21	13	15	25
Guinea pig	53	53	51	51	63	61	100	41	41	43	48	9	16	17	6	18	16
Dog	60	61	57	57	53	51	41	100	56	56	64	16	31	26	18	28	18
Pig	53	54	50	51	51	49	41	56	100	65	56	20	26	25	17	22	15
Cow	54	55	49	46	50	52	43	56	65	100	60	16	25	25	20	21	14
Horse	59	60	59	57	55	51	48	64	56	60	100	20	29	25	8	24	16
Human LY6D	14	17	10	18	14	15	9	16	20	16	20	100	25	30	14	32	28
Human LY6E	27	24	30	27	20	20	16	31	26	25	29	25	100	32	19	17	32
Human LY6H	26	23	25	22	22	21	17	26	25	25	25	30	32	100	16	28	25
Human LY6K	10	10	13	9	9	13	6	18	17	20	8	14	19	16	100	22	18
Human LYPD2	24	24	24	23	25	15	18	28	22	21	24	32	17	28	22	100	31
Human LYNX1	13	13	12	12	14	25	16	18	15	14	16	28	32	25	18	31	100

Numbers show the percentage of amino acid sequence identities

The amino acid sequences for most of the mammalian GPIHBP1 proteins contained 167–184 residues whereas mouse and rat GPIHBP1 contained 225 and 236 amino acids, respectively, with the latter having extended C-terminal sequences (Fig. 1). Previous biochemical and genetic analyses of human and mouse GPIHBP1 (Beigneux et al. 2007; Gin et al. 2007, 2011) have enabled predictions of key residues for these mammalian GPIHBP1 proteins (sequence numbers refer to human GPIHBP1). These included the N-terminus signal peptide (residues 1–20) which participates in the trafficking of GPIHBP1 via the endoplasmic reticulum; two acidic amino acid clusters (residues 25–32 and 41–50) which may contribute to LPL binding within a basic amino acid LPL heparin-binding site region (Sendak and Bensadoun 1998); a conserved Gly56 with an unknown function (Gin et al. 2007); a predominantly conserved N-glycosylation site (Asn78-Leu79-Thr80) which is critical for the movement of GPIHBP1 onto the cell surface (Beigneux et al. 2008); a urokinase plasminogen activator receptor (UPAR)-lymphocyte antigen-6 (LY6) domain which contains 10 conserved cysteine residues (Cys65, Cys68, Cys77, Cys83, Cys89, Cys110, Cys114, Cys130, Cys131 and Cys136) and forms five disulfide bridges within this domain; Gln115 which plays a role in LPL binding to GPIHBP1 (Franssen et al. 2010); and a hydrophobic C-terminal helix domain (residues 160–178) which is replaced by a glycosylphosphatidylinositol anchor (to Gly159) and is responsible for linking GPIHBP1 to the endothelial cell surface (Nosjean et al. 1997; Davies et al. 2010; Fisher 2010). These residues and predicted properties were conserved for all of the mammalian GPIHBP1 sequences examined (Fig. 1) with the exception of the cow GPIHBP1 sequence, which lacked a predicted N-glycosylation site (Beigneux et al. 2008). Predicted N-glycosylation site(s) were also absent in guinea pig, dog and pig GPIHBP1 sequences; whereas human and orangutan GPIHBP1 sequences exhibited two predicted N-glycosylation sites (Asn78-Leu79-Thr80 and Asn82-Cys83-Ser84) (Table 5) although experimental evidence for in vivo N-glycosylation is only available for the first site (Beigneux et al. 2008).

**Table 5:** Predicted N-glycosylation sites for mammalian GPIHBP1 sequences

Mammalian GPIHBP1	Species	Site 1	Site 1 potential	Site 2	Site 2 potential	Site 3	Site 3 potential	Site 4	Site 4 potential	No. of potential sites
Human	*Homo sapiens*	**78NLTQ**	**0.76**	**82NCSH**	**0.61**					2
Orangutan	*Pongo abelii*	**78NLTQ**	**0.76**	**82NCSH**	**0.61**					2
Rhesus	*Macaca mulatta*	**78NLTR**	**0.69**							1
Marmoset	*Callithrix jacchus*	**77NLTQ**	**0.80**							1
Mouse	*Mus musculus*	**76NQTQ**	**0.53**							1
Rat	*Rattus norvegicus*	**84NETQ**	**0.55**							1
Guinea Pig	*Cavia porcellus*	76NQTE	NP					150NGTT	NP	0
Horse	*Equus caballus*	**77NQTQ**	**0.68**			**118NKTV**	**0.70**			2
Cow	*Bos taurus*									0
Dog	*Canis familaris*									0
Pig	*Sus scrofa*									0

Predicted N-glycosylation sites were identified using NetNGlyc 1.0 web tools (http://www.cbs.dtu.dk/services/NetNGlyc/)³², potential for N-glycosylation sites was determined by the web tools (maximum level of 1)

Bold values designate high probability of forming an N-glycosylation site

*N* Asparagine, *L* leucine, *Q* glutamine, *T* threonine, *C* cysteine, *R* arginine, *E* glutamate, *H* histidine, *V* valine, *NP* no prediction for an N-glycosylation site

The human LY6-like sequences examined shared several of the mammalian GPIHBP1 domain regions, including the N-signal peptide region (sequence numbers refer to human LY6D) (residues 1–20); the UPAR-LY6 domain with 10 conserved cysteine residues (Cys23, Cys26, Cys32, Cys38, Cys45, Cys63, Cys67, Cys86, Cys87 and Cys92) forming five disulfide bonds previously reported for LY6-like proteins (Fry et al. 2003; Leath et al. 2007), and the hydrophobic C-terminal helix domain (residues 104–125) which is replaced by a glycosylphosphatidylinositol anchor (predicted to be bound to Asn98). These LY6-like sequences, however, lacked the N-terminal acidic amino acid domain and contained fewer amino acids in the protein region surrounding the UPAR-Ly6 domain (residues 21–96). These sequences also lacked the predominantly conserved N-glycosylation site observed for mammalian GPIHBP1 proteins but contained amidation sites for attaching the glycosylphosphatidylinositol anchor in each case.

### Predicted structures for mammalian GPIHBP1 proteins

Predicted secondary structures for mammalian GPIHBP1 sequences were compared with those predicted for human lymphocyte antigen-6-like proteins (Fig. 1). α-Helix and β-sheet structures for these sequences were similar for several regions with the human LY6-like secondary structures, including the N-terminal signal peptide which contained an extended helical structure; the UPAR-LY6 domain which contained four or five β-sheet structures (designated as β1–β5) within the region for five disulfide bonds; and the C-terminal hydrophobic region, which is removed following GPI-attachment within the endoplasmic reticulum. The distinctive secondary structures observed for mammalian GPIHBP1 sequences were two acidic amino acid α-helical regions which were notably absent in the LY6-like predicted secondary structures.

**Fig. 2:**
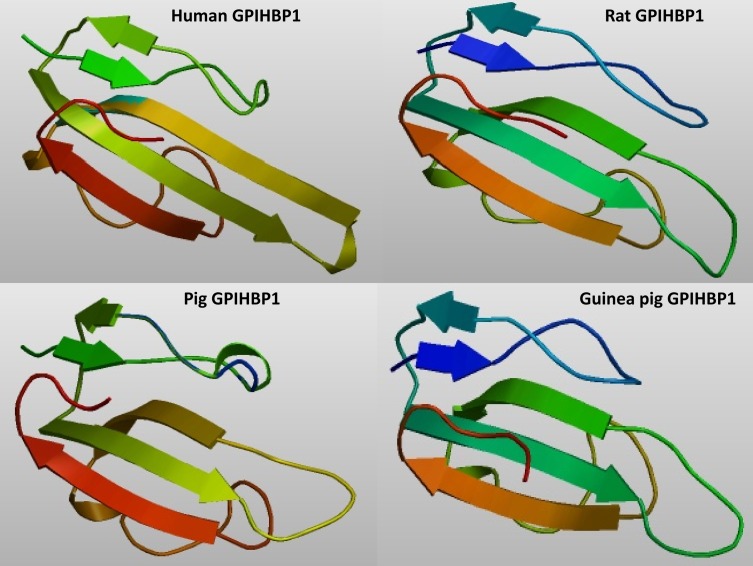
Predicted tertiary structures for the UPAR/Ly6 domain for human, rat, guinea pig and pig GPIHBP1. Predicted GPIHBP1 tertiary structures were obtained using SWISS MODEL methods; the rainbow color code describes the tertiary structures from the N- (*blue*) to C-termini (*red color*) for human, rat, guinea pig and pig GPIHBP1 UPAR/Ly6 domains; *arrows* indicate the directions for β-sheets

Tertiary structures for the members of the LY6 protein family has been reported previously which are characterized by an amino acid motif containing eight or ten cysteine residues arranged in consistent spacing patterns forming four or five disulfide bonds and a three-finger motif which comprised β-pleated sheets predominantly. The predicted secondary structures observed for the human LY6-like proteins (LY6D, LY6E, LY6PD, LY6H, LY6K and LY6NX1) and the mammalian GPIHBP1 protein sequences examined are consistent with the presence of this LY6 protein family motif within these proteins (Fig. 1). Figure 2 describes predicted tertiary structures for human, rat, pig (*Sus scrofa*) and guinea pig (*Cavia porcellus*) GPIHBP1 protein sequences and shows significant similarities to the UPAR-LY6 domain reported for the human CD59 antigen (membrane-bound glycoprotein) (Leath et al. 2007). Five anti-parallel β-sheets are readily apparent in each case, which is consistent with the predictions observed for the human and rat GPIHBP1 proteins shown in the amino acid sequence alignments in Fig. 1. This suggests that the UPAR-LY6 domain secondary and tertiary structures are shared among all GPIHBP1 proteins examined as well as the human LY6-like proteins examined.

The overall structure for mammalian GPIHBP1 may then comprise the two α-helices of acidic amino acids (which bind LPL to GPIHBP1) and the three-fingered β-sheet motif which is covalently linked to the plasma membrane by a glycosylphosphatidylinositol anchor. Recent studies have shown that both motifs are essential for LPL binding and transport and for GPIHBP1 function (Beigneux et al. 2009a, b; Gin et al. 2011).

### Comparative human *GPIHBP1* tissue expression

Beigneux et al. (2009b) have previously examined *Gpihbp1* tissue expression in mouse tissues and reported high levels of expression in heart and adipose tissue, which corresponds with the major distribution for LPL in the body and supports the key role played by this enzyme in lipid metabolism, especially in heart and adipose tissue (Wion et al. 1987; Havel and Kane 2001). Overall, human *GPIHBP1*, and mouse and rat *Gpihbp1* genes were moderately expressed in comparison with the other lymphocyte antigen-like genes being 0.1–0.7 times the average level of gene expression in comparison with human *LY6E* and *LYNX1* genes, which showed expression levels of 4.3 and 1.8 times the average gene, respectively (Table 1). This may reflect a more restricted *GPIHBPI* cellular expression as compared with *LY6*-like genes and/or a more specialized role of GPIHBP1 is being responsible for LPL binding in heart and adipose tissue as compared with the broader and more widely distributed functions of LY6-like proteins as lymphocyte antigens throughout the body.

### Gene locations and exonic structures for mammalian *GPIHBP1* genes and human *LY6*-like genes

Table 1 summarizes the predicted locations for mammalian *GPIHBP1* genes and human *LY6*-like genes based on BLAT interrogations of several mammalian genomes using the reported sequences for human and mouse (Beigneux et al. 2007; Gin et al. 2007, 2011) and the predicted sequences for the other mammalian GPIHBP1 proteins and the UCSC Genome Browser (Kent et al. 2003). Table 2 also presents the predicted locations and other features for mouse, cow and opossum *LY6*-like genes and proteins. The mammalian *GPIHBP1* genes were predominantly transcribed on the positive strand, with the exception of the marmoset and pig genes which were transcribed on the negative strand. Figure 1 summarizes the predicted exonic start sites for mammalian *GPIHBP1* genes with most having 4 coding exons in identical or similar positions to those predicted for the human *GPIHBP1* gene, with the exception of the orangutan *GPIHBP1* gene, which contained an additional exon within the encoding region for the C-terminal sequence. In contrast, the human, mouse, cow and opossum *LY6*-like genes examined contained only 3 coding exons encoded on either the positive or negative strands. These results are indicative of structural similarities between the mammalian *GPIHBP1* and *LY6*-like genes but with the *GPIHBP1* genes possessing an additional exon (exon 2) in each case.

**Fig. 3:**
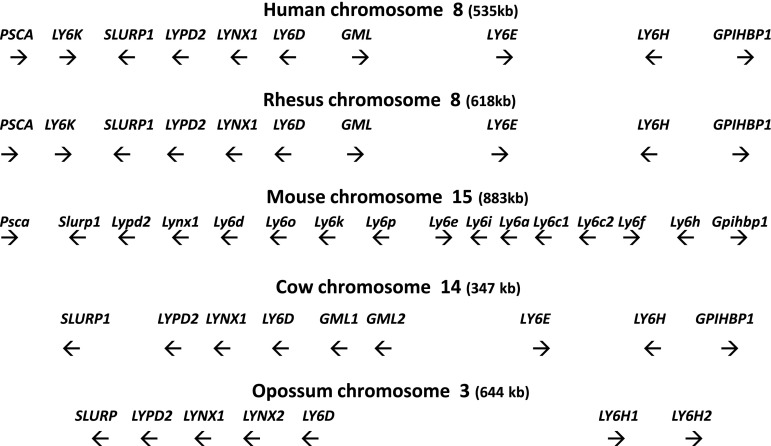
Comparative gene clusters for mammalian *LY6*-like genes. *LY6*-like gene clusters are identified with the size of the cluster (in kilobases) in each case. Individual *LY6*-like genes were identified and positioned using data summarized in Tables 1 and 2. The *arrow* shows the direction for transcription:*right arrow* the positive strand; *left arrow* the negative strand. Note the absence of an identified *GPIHBP1* gene on the opossum genome

Figure 3 summarizes the comparative locations of human, rhesus monkey, mouse, cow and opossum *LY6*-like genes within respective gene clusters. Nine human and rhesus *LY6*-like and the related *GPIHBP1* genes, for example, were localized within 535 or 618 kb gene clusters, respectively, on human and rhesus chromosome 8 whereas 15 mouse *Ly6*-like genes and the *Gpihbp1* gene were co-localized within a 883-kb gene cluster on mouse chromosome 15. Cow and opossum (*Monodelphis domestica*—a marsupial mammal) *LY6*-like genes were also similarly located within respective gene clusters on chromosomes 14 and 3, respectively, although in each case, there were fewer *LY6*-like genes identified in comparison with human and rhesus genomes, and particularly the mouse genome. Of special interest to this current study, however, is the absence of an identified opossum *GPIHBP1*-like gene and the presence of two predicted opossum *LY6H*-like genes on chromosome 3 of the opossum genome. For each of the mammalian genomes examined (human, rhesus monkey, mouse, cow and opossum), there were similarities in *LY6*-like gene order: *LYPD2*-*LYNX1*-*LY6D*-*LY6E*-*LY6H*-*GPIHBP1*, but with *GPIHBP1* being undetected in the case of the opossum genome.

**Fig. 4:**
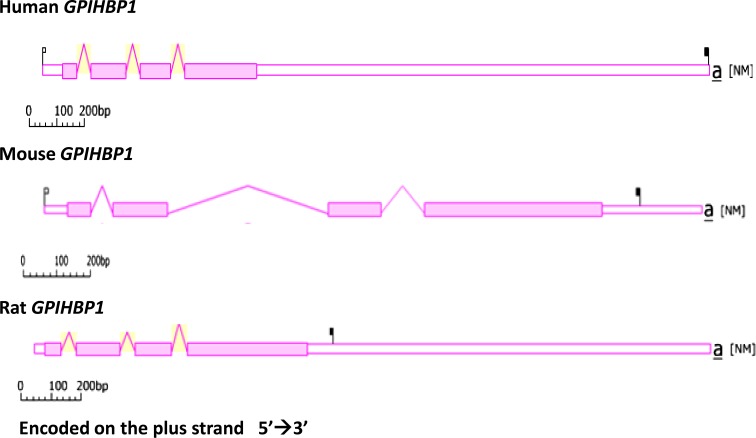
Gene and mRNA structures for the human, mouse and rat *GPIHBP1* genes. Derived from the AceView website http://www.ncbi.nlm.nih.gov/IEB/Research/Acembly/ (Thierry-Mieg and Thierry-Mieg 2006); mature isoform variants (*a*) are shown with capped 5′- and 3′-ends for the predicted mRNA sequences. NM refers to the NCBI reference sequence. Exons are in *shaded pink*; untranslated 5′- and 3′ sequences are in *open pink*, introns are represented as *pink lines* joining exons, the directions for transcription are shown as 5′→3′, sizes of mRNA sequences are shown in kilobases (kb)

Figure 4 shows the predicted structures of mRNAs for human, mouse and rat *GPIHBP1* transcripts (Thierry-Mieg and Thierry-Mieg 2006) which were 2.3–3.1 kbs in length with three introns and four exons present and in each case, an extended 3′-untranslated region (UTR) was observed.

### Evolutionary appearance of the *GPIHBP1* gene in mammalian genomes

**Fig. 5:**
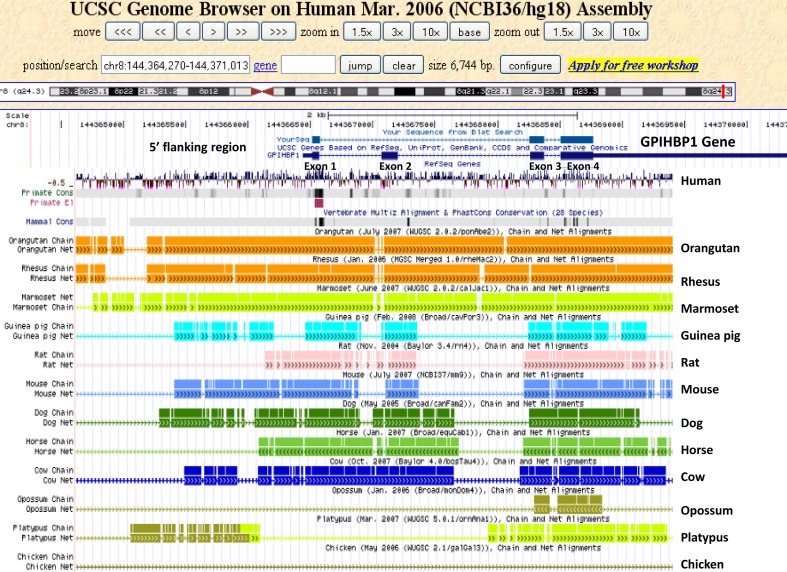
Comparative sequences for mammalian 5′-flanking, 5′-untranslated and coding regions for the *GPIHBP1* genes. Derived from the UCSC Genome Browser using the Comparative Genomics track to examine alignments and evolutionary conservation of *GPIHBP1* gene sequences; genomic sequences aligned for this study included primate (human, orangutan, rhesus and marmoset), non-primate eutherian mammal (mouse, rat, guinea pig, dog, horse and cow), a marsupial (opossum), a monotreme (platypus) and bird species (chicken); conservation measures were based on conserved sequences across all of these species in the alignments which included the 5′-flanking, 5′-untranslated, exons, introns and 3′-untranslated regions for the *GPIHBP1* gene; regions of sequence identity are shaded in *different colors* for different species

Figure 5 shows a UCSC Genome Browser Comparative Genomics track that shows evolutionary conservation and alignments of the nucleotide sequences for the human *GPIHBP1* gene, including the 5′-flanking, 5′-untranslated, intronic, exonic and 3′-untranslated regions of this gene, with the corresponding sequences for 12 mammalian and bird genomes, including 4 primates (e.g., rhesus), 6 non-primate eutherian mammals (e.g., mouse, rat), a marsupial (opossum), a monotreme (platypus) and a bird species (chicken). Extensive conservation was observed among these *GPIHBP1* genomic sequences for the eutherian mammalian genomes, particularly for the primate species but also for the exonic and 5′-flanking regions for all eutherian genomes examined. An examination of non-synonymous (ns) single nucleotide polymorphisms (SNPs) within the human genome supported this conclusion of *GPIHBP1* conservation with this gene containing only a single ns-SNP within exon 1. In contrast with the eutherian mammalian genomes examined, the opossum (marsupial mammal) genome lacked conserved sequences within the 5′-flanking and exon 1 and 2 regions, but showed some genomic sequence conservation within the exon 3 and exon 4 regions. The platypus (monotreme mammal) exhibited conserved *GPIHBP1* gene sequences within the 5′-flanking and exon 3 and 4 regions but showed no conservation of other sections of this gene, and lacked exon 1 and 2 conserved sequences. In addition, the chicken (bird) genomic sequence showed no significant conservation of any region of the *GPIHBP1* gene, which is consistent with BLAT analyses undertaken using mammalian GPIHBP1 protein sequences which failed to identify a *GPIHBP1* gene in this bird genome. It would appear that *GPIHBP1* has only recently evolved during mammalian evolution and that the functional gene is present only in eutherian mammalian genomes.

### Phylogeny and divergence of mammalian GPIHBP1 and LY6-like sequences

**Fig. 6:**
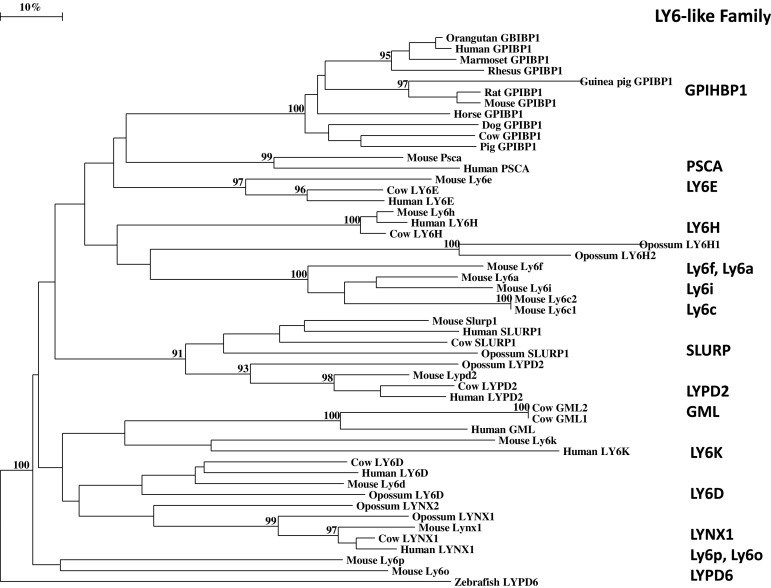
Phylogenetic tree of mammalian GPIHBP1 and other LY6-like sequences. The tree is labeled with the gene name and the name of the animal and is ‘rooted’ with the zebrafish (*Danio rerio*) LY6PD sequence. Note the major cluster for the mammalian GPIHBP1 sequences and several major groups of the other LY6-like sequences: LYNX1, LY6D, LY6H, SLURP1, LYPD2, PSCA, LT6E, LY6K, and GML. A genetic distance scale is shown (% amino acid substitutions). The number of times a clade (sequences common to a node or branch) occurred in the bootstrap replicates are shown. Only replicate values of 90 or more which are highly significant are shown with 100 bootstrap replicates performed in each case

A phylogenetic tree (Fig. 6) was calculated by the progressive alignment of 11 mammalian GPIHBP1 amino acid sequences with human, mouse, cow and opossum LY6-like sequences which was ‘rooted’ with the zebrafish (*Danio rerio*) LYPD6 sequence (Tables 1, 2). The phylogram showed clustering of the sequences into groups which were consistent with their evolutionary relatedness as well as distinct groups for mammalian GPIHBP1 and LY6-like sequences, which were distinct from the zebrafish LYPD6 sequence. In addition, the mammalian LY6-like sequences were further subdivided into groups, including PSCA, LYNX1, LY6D, LY6H, SLURP1, LYPD2, LY6E, LY6K, GML and a group of mouse Ly6-like sequences (designated as Ly6a, Ly6c1, Ly6c2, Ly6f and Ly6i). These groups were significantly different from each other (with bootstrap values >90) and have apparently evolved as distinct genes and proteins during mammalian evolution. Moreover, it is apparent that *GPIHBP1* is a distinct but related *LY6*-like gene which has appeared early in eutherian mammalian evolution.

### Hypothesis: proposed mechanism for the evolutionary appearance of *GPIHBP1* in eutherian mammals

**Fig. 7:**
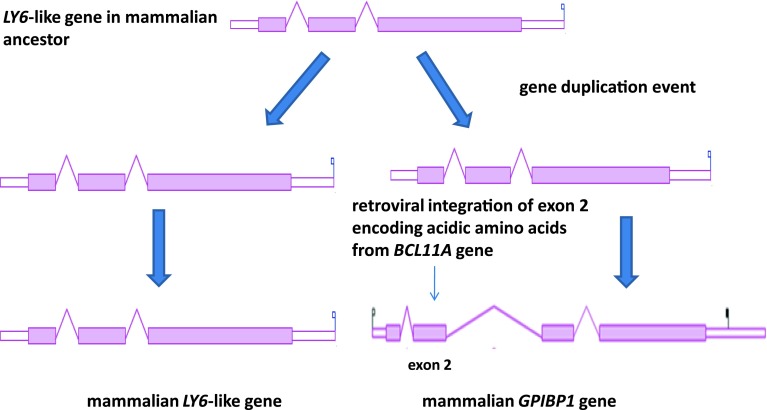
Proposal for generating the *GPIHBP1* gene during eutherian mammalian evolution. This hypothesis is for a two-step process for generating the *GPIHBP1* gene: (1) a *LY6*-like gene duplication event in a common ancestor for eutherian mammals; and (2) retroviral transfer of a region of the *BCL11A* gene in the ancestral genome encoding acidic amino acids generating a *GPIHBP1*-like gene containing a new exon

A search was undertaken for a potential gene ‘donor’ for the exon encoding the acidic amino acid motif contained within the mammalian *GPIHBP1* gene using BLAT to interrogate the human genome with the known nucleotide sequence for exon 2 of the human *GPIHBP1* gene (Kent et al. 2003). A region of the human *BCL11A* gene (encoding acidic residues 484–504 of human B-cell CLL/lymphoma 11A) was identified which encoded an extended sequence of acidic amino acids comparable to amino acid residues 25–50 (corresponding to residues encoded by exon 2 of human *GPIHBP1*) in the human GPIHBP1 sequence. Supplementary Fig. 1 shows an alignment of this region for representative vertebrate BCL11A acidic amino acid sequences with several mammalian GPIHBP1 exon 2 sequences. Similarities in acidic amino acid sequences are apparent although each protein exhibited a distinctive conservation pattern. It may be noted that the *BCL11A* gene and protein can be traced back to reptiles and fish in vertebrates (Table 3) whereas *GPIHBP1* has been only reported in eutherian mammals (Table 1). Previous studies have shown that the mouse *Bcl11a* gene encodes a C2H2-type zinc-finger protein which is a common site of retroviral integration in myeloid leukemia and functions as a myeloid and B-cell proto-oncogene (Nakamura et al. 2000) and may serve as a candidate gene for the transfer and integration of the acidic amino acid encoding ‘motif’ into the mammalian *GPIHBP1* gene. A hypothesis concerning the evolutionary appearance of the ‘ancestral’ eutherian mammalian *GPIHBP1* gene is presented in Fig. 7.

Step 1 An *LY6*-like gene within a common ancestor to eutherian mammals underwent a tandem duplication event generating two closely related *LY6*-like genes. It may be noted that the opossum genome contains similar *LY6H* genes (designated as *LY6H1* and *LY6H2*) which are closely localized on opossum chromosome 3 (Fig. 3) and form a distinct opossum *LY6*-like group following CLUSTAL analysis (Fig. 6); and
Step 2 Retroviral integration of the acidic amino acid encoding ‘motif’ of the ancestral *BCL11A* gene may have occurred in one of the duplicated *LY6*-like genes (potentially a *LY6H*-like gene or another *LY6*-like gene) resulting in the addition of an exon (exon 2) which during the subsequent evolution generates an ancestral eutherian mammalian *GPIHBP1*-like gene and protein which is retained throughout subsequent eutherian mammalian evolution.

## Conclusions

The results of the present study indicate that the mammalian *GPIHBP1* gene and encoded protein recently reported represents a distinct family of lymphocyte antigen-6 (*LY6*)-related gene and protein which shares key conserved sequences and functions with other *LY6*-like genes and proteins previously studied (Brakenoff et al. 1995; Capone et al. 1996; Clark et al. 2003; Horie et al. 1998; Ishikawa et al. 2007). GPIHBP1 is encoded by a single gene among the mammalian genomes studied which is localized within a *LY6*-like gene cluster (~500 kbs) on human chromosome 8 and usually contained 4 coding exons. Predicted secondary structures for mammalian GPIHBP1 proteins showed a strong similarity with other LY6-like proteins in a number of domains, including the N-terminal signal peptide region, the UPAR-LY6 domain and in having a highly hydrophobic C-terminal helical sequence, which is removed in the endoplasmic reticulum during the formation of the glycosylphosphatidylinositol anchor. In contrast, however, all mammalian GPIHBP1 proteins contained two high acidic amino acid regions, which have been proposed to play a role in binding LPL (Beigneux et al. 2007; Gin et al. 2007, 2011). Predicted secondary and tertiary structures of the UPAR-LY6 mammalian GPIHBP1 domain showed a strong resemblance to the corresponding region for the human CD59 antigen structure (Leath et al. 2007) with five anti-parallel β-sheets. Comparative studies of 12 mammalian *GPIHBP1* genomic sequences indicated that this gene has appeared during eutherian mammalian evolution with conserved genomic sequences observed for all eutherian mammalian genomes examined. In contrast, *GPIHBP1* gene sequences were absent from the chicken genome or were seen only in part for the monotreme and marsupial genomes examined. It is proposed that the *GPIHBP1* gene has appeared early in mammalian evolution following a tandem gene duplication event of one of the *LY6* genes and the subsequent retroviral integration of exon 2 encoding the acidic amino acid ‘motif’.

## Electronic supplementary material

Below is the link to the electronic supplementary material.

Supplementary Fig. 1: Alignments for Acidic Amino Acid Sequence Regions for Vertebrate BCL11A and Mammalian GPIHBP1 Sequences

See Tables 1 and 3 for sources of glycosylphosphatidylinositol-anchored high-density lipoprotein-binding protein 1 (GPIHBP1) and vertebrate *BCL11A* gene (encoding B-cell CLL/lymphoma 11A) sequences; * shows identical residues for proteins; : similar alternate residues;. dissimilar alternate residues; acidic amino acids are in **blue**; basic amino acid residues in pink; hydrophobic amino acids in **red**; and hydrophilic amino acids in **green**

Supplementary material 1 (PPT 139 kb)
