# Supplementary figures and images for: Comparative studies of glycosylphosphatidylinositol-anchored high-density lipoprotein-binding protein 1: evidence for a eutherian mammalian origin for the GPIHBP1 gene from an LY6-like gene
Source: 3 Biotech. 2011 Oct 18;2(1):37–52. doi: 10.1007/s13205-011-0026-4 (PMC3339605; doi:10.1007/s13205-011-0026-4)

## Slide 1
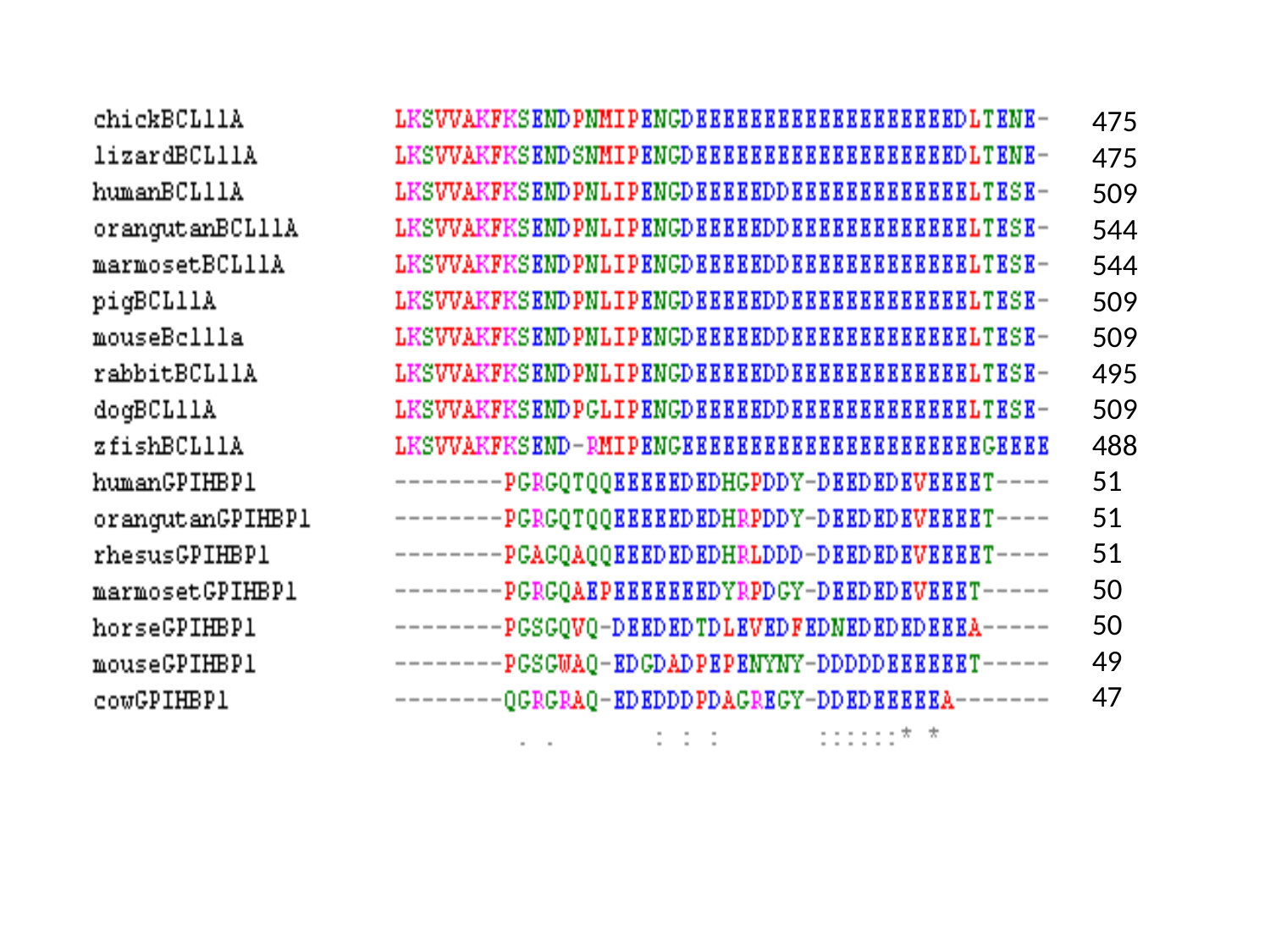

475
475
509
544
544
509
509
495
509
488
51
51
51
50
50
49
47

Supplement: Supplementary file 1 — Supplementary material 1 (PPT 139 kb) [file 13205_2011_26_MOESM1_ESM.ppt]
